# A novel method for noninvasive bioelectric measurement utilizing conductivity of seawater

**DOI:** 10.1038/s41598-021-86295-y

**Published:** 2021-03-29

**Authors:** Tsunemasa Saiki, Yukako Takizawa, Koji Murai, Ryuhei Okuno, Masakazu Arima

**Affiliations:** 1 Materials and Analysis Department, Hyogo Prefectural Institute of Technology, 3-1-12, Yukihira-cho, Suma-ku, Kobe, Hyogo, 654-0037 Japan; 2 Graduate School of Engineering, University of Hyogo, 2167, Shosha, Himeji, Hyogo, 671-2201 Japan; 3 Manufacturing Technology Department, Hyogo Prefectural Institute of Technology, 3-1-12, Yukihira-cho, Suma-ku, Kobe, Hyogo, 654-0037 Japan; 4 Department of Maritime Systems Engineering, Tokyo University of Marine Science and Technology, 2-1-6, Eechujima, Koto-ku, Tokyo, 135-8533 Japan; 5 Faculty of Science and Engineering, Setsunan University, 17-8, Ikeda-nakamachi, Neyagawa, Osaka, 572-8508 Japan; 6 Graduate School of Engineering, Osaka Prefecture University, 1-1, Gakuen-cho, Naka-ku, Sakai, Osaka, 599-8531 Japan

**Keywords:** Electrophysiology, Biomedical engineering, Health care

## Abstract

A novel method of noninvasive bioelectric measurement that utilizes the conductivity of seawater covering a person’s whole body is proposed. Concretely, a conductor used as a common electrode is sunk into the seawater, and four special bioelectrodes isolated from the seawater are attached at measurement points on the body. Bioelectric signals generated between the common electrode and special bioelectrodes are then measured. To verify the effectiveness of the proposed method, bioelectric signals of six participants immersed in a bathtub filled with seawater were experimentally measured. The measurement results revealed that the proposed method enables multipoint bioelectric measurement using about half the number of bioelectrodes used by the conventional method on land, and a plurality of bioelectric phenomena can be observed at one measurement point. It was also revealed that compared with the conventional method, the proposed method significantly reduces external electrical noise included in the bioelectric signals by exploiting the shielding effect of seawater. If simple bioelectric measurements in seawater were possible in the manner described above, not only people such as scuba divers but also precious animals living in the sea could be noninvasively treated as measurement subjects.

## Introduction

Since the early twentieth century (1901) when Willem Einthoven measured an electrocardiogram (ECG) with a galvanometer, noninvasive bioelectric measurements have been mainly performed on land^1,2^. Presently, noninvasive bioelectric measurements, such as ECGs, electromyograms (EMGs), and electroencephalograms (EEGs), are widely used for not only medical diagnoses of patients but also for evaluating motor skills of athletes^3,4^ and monitoring heath of elderly people^5^.

As one of those measurements, namely, unique bioelectric measurements in water, EMGs of swimmers have been measured to determine how they efficiently swim^6^. In such studies, EMGs or ECGs were generally measured by attaching and isolating bioelectrodes on areas of skin sealed from water with waterproof tape or some other means^6–13^. Thus, the principle of bioelectric measurement by such an isolation method in water is the same as the conventional principle of that on land, and the same number of bioelectrodes is needed in water as that needed on land. As a study of bioelectric measurement using a different principle from the conventional one, ECG measurement for monitoring the health of people soaking in a bath has been reported^14^. In that study, multiple bioelectrodes were not attached to the person’s body; instead, they were installed on the sidewalls of the bathtub, and ECGs were successfully measured by using the property of freshwater (which is not a perfect insulator). A non-isolation method based on this principle can be used to measure EMGs in freshwater^15–17^. Interestingly, the EMG potentials could be observed at bioelectrodes located about one centimeter away from a person’s skin^17^.

In the meantime, since most diving is done in the sea, there is a strong demand for bioelectric measurement for people in seawater as well as freshwater. This is because very little is known about the physical and physiological characteristics of people (divers) in a dangerous environment where the diver is subjected to water pressure and has no air to breathe around them. Under those circumstances, it is desirable to monitor the safety of the divers working in seawater. Incidentally, as for diving accidents in Japan (a country surrounded by the sea), the rate of death or missing is very high; namely, it makes up 25 to 40% of the total accidents, depending on the year^18^. Therefore, the above-mentioned isolation method, i.e., preventing a short circuit between the bioelectrodes via seawater (with conductivity), can be used to measure bioelectric signals^17,19^. Naturally, the principle of this measurement method in seawater is basically the same as that of the conventional method on land.

Taking a hint from a famous experiment in which Willem Einthoven measured an ECG of a person by immersing the person’s hands and one foot in containers filled with salt water, we recently proposed a method for invasive bioelectric measurement that uses seawater covering an entire fish’s body as one huge electrode^20^. This measurement method is based on a different principle from that of the isolation method in seawater. As for this method, people could not be treated as measurement subjects ethically, because it is necessary to insert bioelectrodes into their bodies. Therefore, in the present study, we propose a novel method for noninvasive bioelectric measurement as an improvement on the previous method.

**Figure 1:**
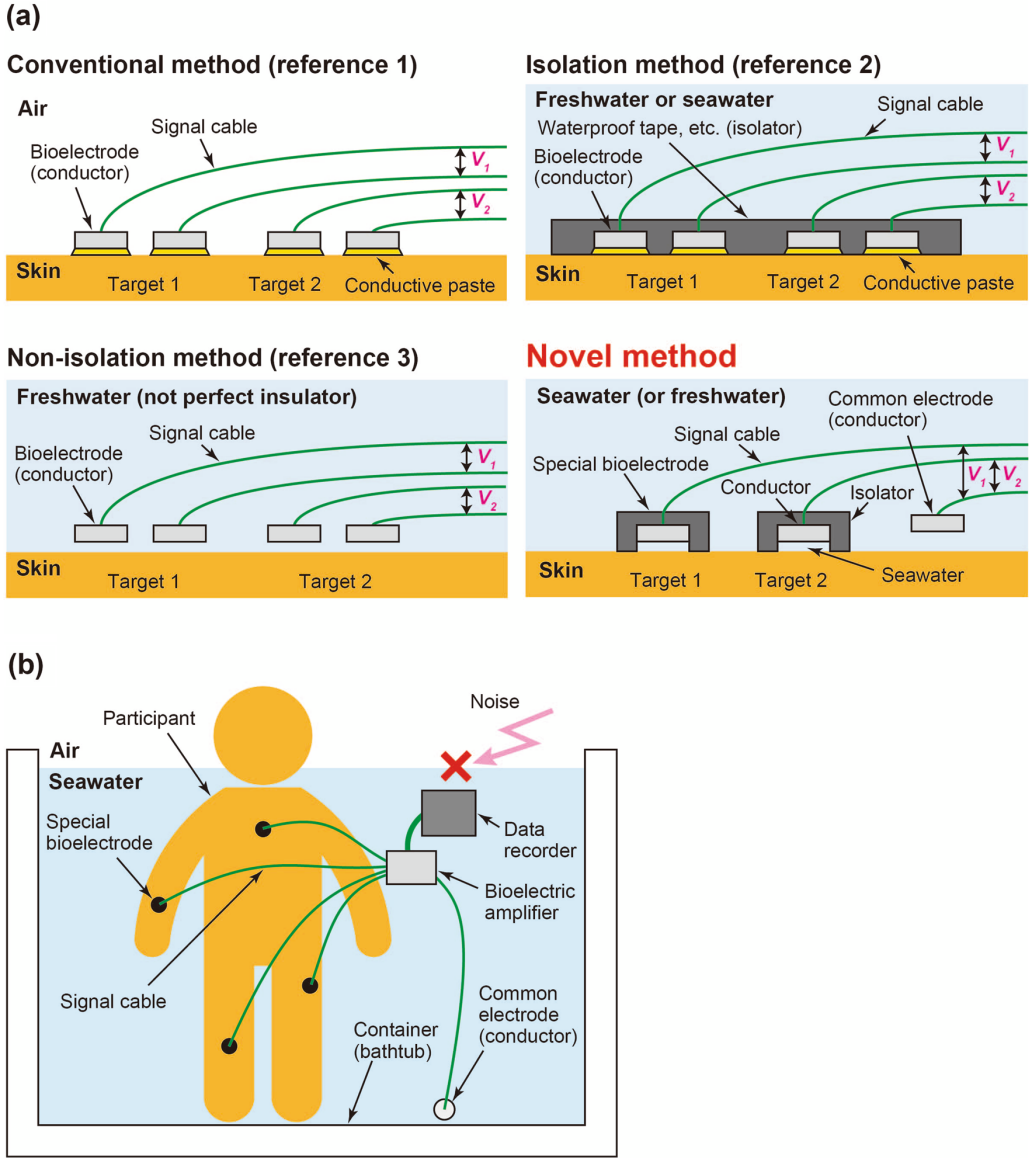
Novel method for noninvasive bioelectric measurement in seawater: (**a**) principle and (**b**) system.

As for the novel method (Fig. 1), special bioelectrodes isolated from seawater are attached at measurement points on the person’s skin, and one conductor (used as a common electrode) is sunk into the seawater. Voltage signals generated between the common electrode and special bioelectrodes are then measured. The number of necessary bioelectrodes is therefore reduced by about half, and the amount of labor needed for attaching the bioelectrodes is also reduced. Moreover, all measurement apparatuses and the person’s body are electrically shielded by seawater, so the bioelectric signal can be measured in an environment containing very little external electrical noise. If a simple noninvasive method for a highly effective bioelectric measurement in seawater were established, it would enable not only safety monitoring of scuba divers but also light-load rehabilitation and training (using underwater weightlessness) for patients and athletes as well as health management and ecological investigation of precious marine animals, such as the species *rhincodon typus* and *orcinus orca*, kept at ocean aquariums.

In this paper, the measurement system used to verify the proposed method is first described in detail. Next, the experimental procedure for bioelectric measurement using this system is described, and the results of the experimental bioelectric measurements are presented. Finally, on the basis of the obtained results, the above-mentioned effectiveness of the proposed method is discussed.

## Measurement system

A system for conducting bioelectric measurements in seawater (Fig. 1b) consists of three parts: electrodes (a common electrode and four special bioelectrodes) for detecting electrical signals generated in vivo, a bioelectric amplifier for amplifying the detected electrical signals, and a data recorder for recording the amplified signals.

**Figure 2:**
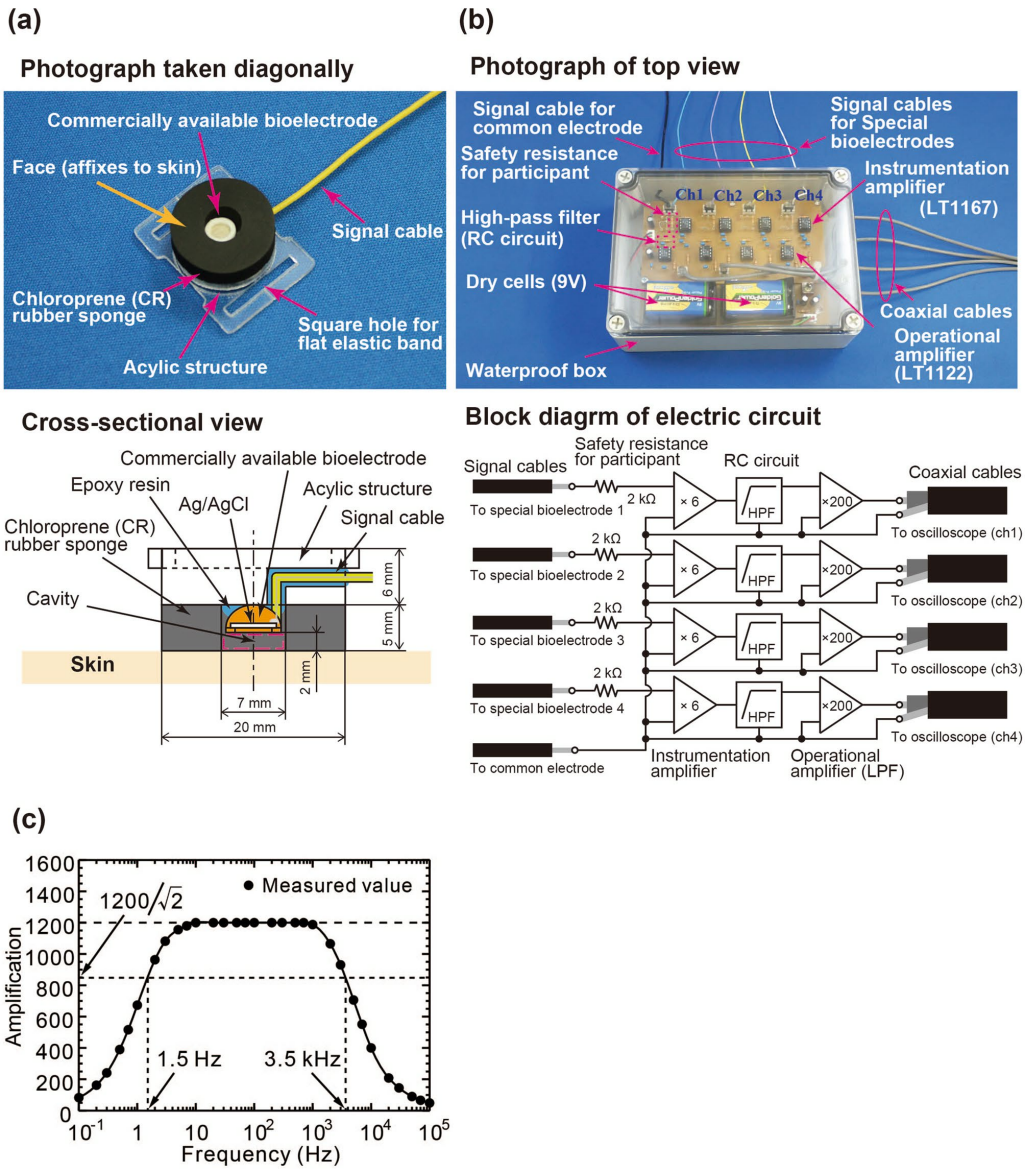
(**a**) Special bioelectrode isolated from seawater, (**b**) fabricated bioelectric amplifier, and (**c**) frequency characteristics of fabricated bioelectric amplifier.

The common electrode is a brass plate (100 × 50 × 5 mm^3^) sunk into approximately 300 L of simulant seawater (electrical conductivity: 5.3 S/m)^21^ in a bathtub (134-cm length × 69-cm width × 50-cm depth). The special bioelectrodes were fabricated by fixing a piece of annular chloroprene sponge rubber (20-mm outside diameter, 7-mm inside diameter, and 5-mm thickness; CSC2 hardness: 39) on an acrylic structure by silicon elastic adhesive (Cemedine Co., Ltd., Super X), embedding a commercially available Ag/AgCl electrode (Unique Medical Co., Ltd., EPA-12, 6-mm diameter, and 3-mm thickness) in the hole of the piece of sponge rubber, and fixing the Ag/AgCl electrode with epoxy adhesive (Nichiban Co., Ltd., Araldite Rapid) (Fig. 2a). The special bioelectrodes were attached to a participant immersed in seawater by an elastic band (15-mm width) passed through the holes of the acrylic structure. As a result of this configuration, a cavity was created in front of the Ag/AgCl electrode and filled with seawater, and the seawater in the cavity was isolated from the outside seawater by the special bioelectrodes and the participant’s skin. Incidentally, in the case of the conventional method for bioelectric measurement, the cavity seawater has the same effect as conductive paste in regard to reducing the contact resistance between a bioelectrode and a person’s skin (Fig. 1a).

A four-channel bioelectric amplifier was designed and fabricated in-house (Fig. 2b). To allow immersion in seawater, the electric circuit of the amplifier was housed in a waterproof polycarbonate box (Takachi Electrics Enclosure Co., Ltd., SPCP131806T: 125-mm width × 175-mm length × 60-mm height). In the amplifier, a bioelectric signal input from the common electrode and each special bioelectrode is amplified six times by an instrumentation amplifier IC (Analog Devices, Inc., LT1167). Incidentally, one of the two input terminals of the instrumentation amplifier IC is connected to the common electrode and also to the ground of the oscilloscope through the ground of the bioelectric amplifier. Next, to suppress baseline variations depending on motion artifacts, the low-frequency components of the amplified signal were attenuated by the RC high-pass filter. Finally, the amplified and filtered signal was additionally amplified 200 times by an operational amplifier IC (Analog Devices, Inc., LT1122). At the same time as the amplification, the high-frequency component was attenuated by the frequency characteristic of the operational amplifier IC.

**Figure 3:**
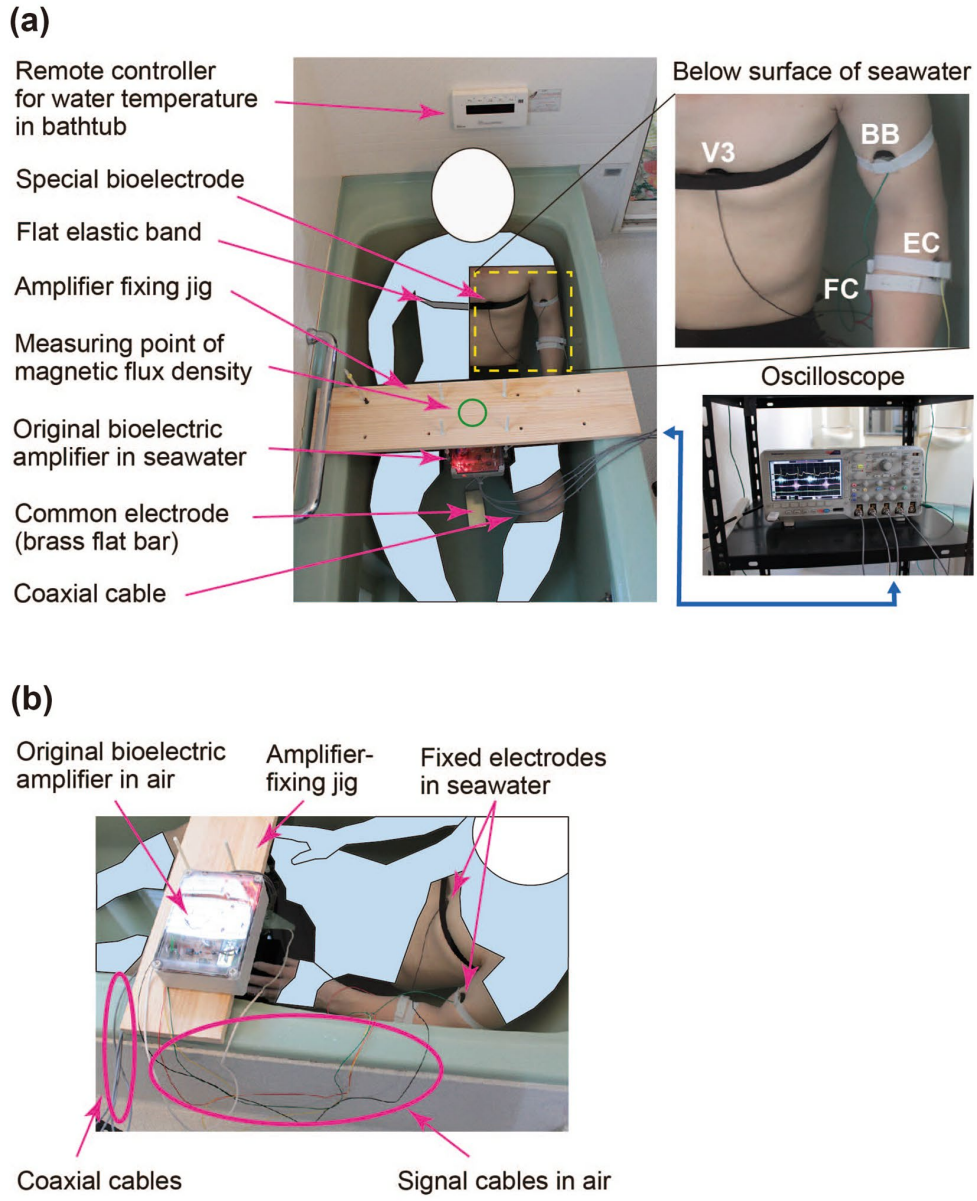
(**a**) Participant during bioelectric measurement in seawater and (**b**) bioelectric amplifier and signal cables lifted out of the seawater for investigating the shielding effect of the seawater.

The frequency characteristic of the fabricated bioelectric amplifier was experimentally investigated as shown in Fig. 2c. In this experiment, an arbitrary frequency-sine-wave signal with 100-µV_p-p_ amplitude generated by a function generator is input into the fabricated bioelectric amplifier directly, and the output-voltage signal from the bioelectric amplifier was observed by an oscilloscope. The amplifier’s amplification factor for each frequency was then obtained. The figure shows that the bioelectric amplifier can remove the low- and high-frequency components of the original bioelectric signal at cut-off frequencies of 1.5 Hz and 3.5 kHz, respectively, and the bioelectric signal can be amplified 1200 times. Moreover, the amplifier’s amplification factor in seawater was also obtained by connecting the function generator between the bioelectric amplifier and a special bioelectrode, and then sinking the special bioelectrode and a brass plate directly connected to the bioelectric amplifier at both ends of the bathtub filled with seawater (Fig. 3a in “Experimental procedure”). As a result, as shown in Fig. 2c, the frequency characteristic of the fabricated bioelectric amplifier is not affected by the use of seawater in the measurement system.

However, a metal shield case and a notch-filter circuit were not adopted, so the fabricated amplifier was much more susceptible to external noise than a commercial bioelectric amplifier. As for this susceptibility, the electrical-shielding effect of seawater can be precisely investigated by locating the fabricated amplifier in seawater and out of seawater (in air). In addition, in consideration of suppression of internal-noise generation and safety for the participant, the fabricated amplifier is driven by a battery (± 9 V). Incidentally, even if the bioelectric amplifier fails, electric current flowing in the human body can be controlled and reduced by a safety resistor (2 kΩ) mounted on the electric circuit.

A commercially available oscilloscope (Tektronix Inc., MSO2024) was used as a data recorder for the amplified bioelectric signals. Incidentally, input impedance of the oscilloscope is 1 MΩ. In consideration of its operability and being non-waterproof, the oscilloscope was located in air away from the seawater.

## Experimental procedure

Bioelectric measurements were conducted on six healthy Japanese participants (A: 49-year-old male; B: 22-year-old male; C: 22-year-old male; D: 49-year-old female; E: 53-year-old female; and F: 26-year-old female). Before the measurement, the participant, in the Fowler’s position in the bathtub, was immersed in the simulant seawater at 35 ± 1 °C up to the neck, as shown in Fig. 3a. Then, the four special bioelectrodes were attached at the V3 position of a standard 12-lead ECG, the center position of the left biceps brachii (BB), the center position of the left flexor carpi (FC), and the center position of the left extensor carpi (EC) of the participant. Next, the brass plate (the common electrode) was sunk at approximately the center of the bathtub bottom, i.e., between the lower part of the participant’s thighs. At that time, if the special bioelectrode was properly attached to the participant’s skin, i.e., the inner seawater of the special bioelectrode was separated from the outer seawater, the resistance between the common electrode and the special bioelectrode became several kiloohms or more and several-dozen kiloohms or less regardless of the special bioelectrode’s position and participant (Table 1). Thereafter, the bioelectric amplifier was installed in the seawater (approximately 10 cm between the water surface and the upper surface of the amplifier) by using an in-house-fabricated jig. Incidentally, the jig was made of wood and resin (through which electromagnetic waves pass), and magnetic flux density was measured with an electromagnetic field meter (AlphaLab, Inc., TF2) as about 10 nT at the center of the jig.

**Table 1:** Resistance between common electrode and special bioelectrode.

Participant	Resistance (kΩ)			
	V3	BB	FC	EC
A	11.8	10.6	7.7	5.4
B	9.3	9.2	10.4	9.5
C	15.8	8.3	8.4	9.3
D	15.4	18.9	13.2	21.7
E	10.5	19.1	8.5	7.6
F	19.4	16.1	9.6	7.5

*Note*: All resistance values were measured by a specialized resistance meter (Nihonsanteku Co., MaP811).

After the above-described preparations for measurement were completed, bioelectric signals when the participant was in motion and at rest were recorded by an oscilloscope with sampling interval of 16 μs. As for the motion, the participant repeatedly moved one wrist at the maximum angle in the palmar and dorsi-flexion directions at intervals of 1 s. At that time, the participant extended all fingers of the left hand, and kept the other part in resting state. After the measurement, to examine the shielding effect of seawater, the bioelectric amplifier and signal cables were lifted out of the seawater in the bathtub as shown in Fig. 3b, and the bioelectric signals of the participant at rest were also recorded. Note that to perform bioelectric measurements with this system, the entire length of the signal cables could not be lifted out of the seawater because it was necessary to keep the brass plate for the common electrode and the special bioelectrodes under the seawater.

Conforming to the principles of the Declaration of Helsinki and the Japanese Ethical Guidelines for Medical and Health Research Involving Human Subjects, this study was approved by the Ethics Committee of Osaka Prefecture University (March 5, 2019). The measurements were conducted after obtaining informed consent from the participants by letter. The informed consent included the participant’s agreement to publish a photograph from which personally identifiable information was removed (Fig. 3).

## Results and discussion

**Figure 4:**
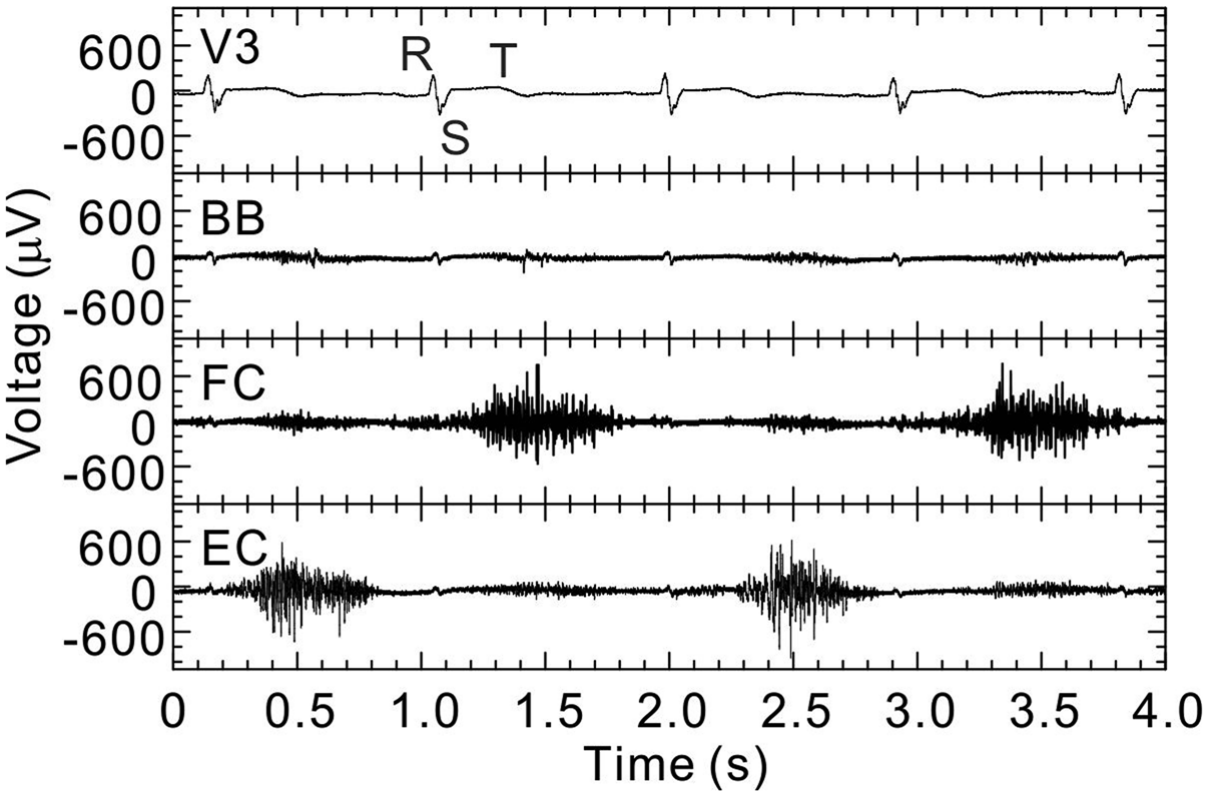
Examples of bioelectric signals when the participant is moving the wrist (participant A). Measurement accuracies of the waveforms at thorax (V3), biceps brachii (BB), flexor carpi (FC), and extensor carpi (EC) were 5.3, 15.6, 15.6, and 15.6 µV, respectively.

Examples of bioelectric signals obtained from the thorax (V3), biceps brachii (BB), flexor carpi (FC), and extensor carpi (EC) when the participant was moving his wrist repeatedly are shown in Fig. 4. These bioelectric signals were obtained from participant A. As shown in the first (top) bioelectric-signal waveform measured at the chest (V3), the R, S, and T waves of the ECG are clearly observed. It was determined from the R-R interval that the heart rate of participant A was about 60 bpm. Next, as shown in the third and fourth bioelectric-signal waveforms for the lower arm, rapidly changing voltages were alternately observed at FC and EC about every second. These voltages are EMG potentials corresponding to the palmar flexion and dorsiflexion of participant A. Such ECG and EMG potentials were similarly observed in the other participants’ (B to F) bioelectric signals. From the above-mentioned results, it is concluded that by attaching only one bioelectrode at each measurement point, bioelectric measurements can be performed in seawater. Therefore, the proposed method applied in seawater has the advantage of being able to measure bioelectric signals with about half the number of bioelectrodes as used by the conventional method in air. Incidentally, as for the measurement system, when the participants repeatedly inclined their upper bodies forward about 20 degree, the baselines of these bioelectric signals (V3, BB, FC, and EC) varied several hundred microvolts with the body movements. Since the baseline variations were smaller and slower than those of the R wave and myoelectric potential, it is clear that heart and muscle activities can be observed easily from the ECGs and EMGs, respectively, measured by using the proposed method.

**Figure 5:**
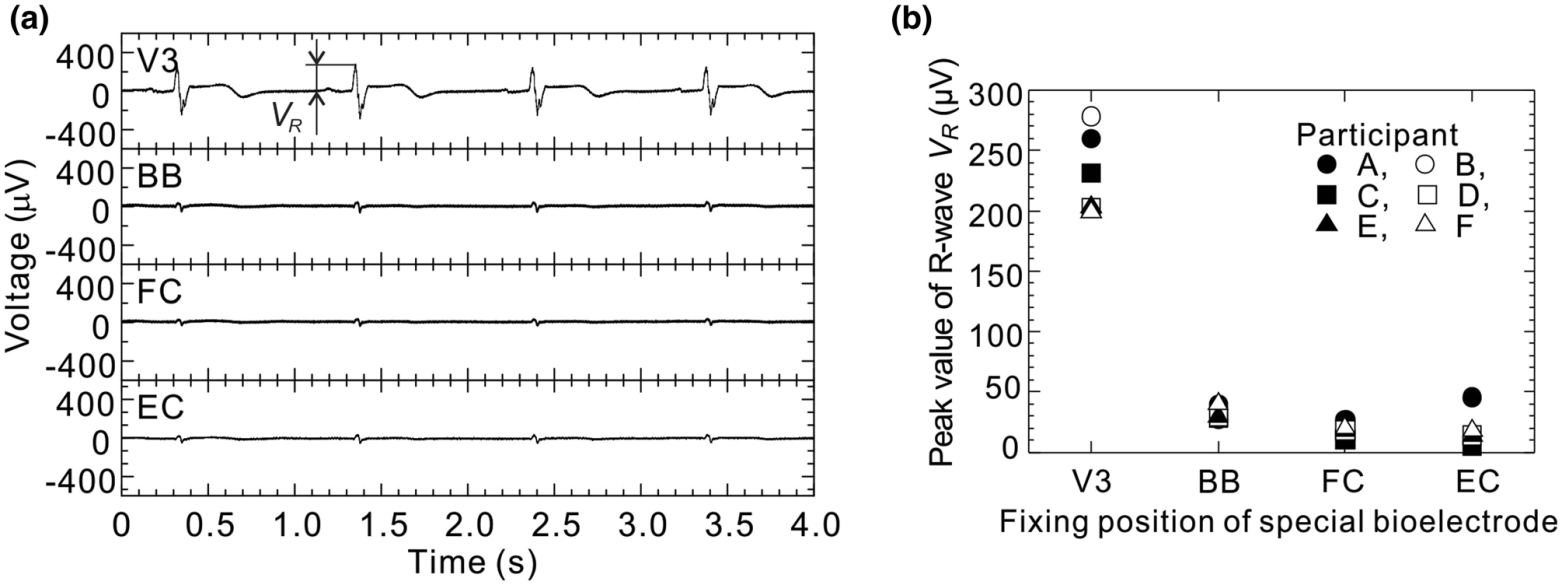
(**a**) Examples of bioelectric signals in rest state (participant A) and (**b**) peak value of R-wave of ECG obtained at each measurement point. Measurement accuracies of the waveforms at thorax (V3), biceps brachii (BB), flexor carpi (FC), and extensor carpi (EC) were 5.3, 2.6, 2.6, and 2.6 µV, respectively.

Examples of bioelectric signals obtained while the participant was in rest state are shown in Fig. 5a. As shown in the figure, although smaller than the ECG potential at the chest (V3), ECG potentials at the arms (BB, FC, and EC) are also observed. Therefore, the R-wave’s peak values (*V*_*R*_) of the ECG bioelectric signals obtained from all the participants are summarized for each measurement point in Fig. 5b. As shown in this figure, *V*_*R*_ at the arms (BB, FC, and EC) of all the participants is considerably smaller than *V*_*R*_ at their chests (V3). Furthermore, when this figure is looked at in detail, it is clear that except for *V*_*R*_ at EC of participant A, *V*_*R*_ of each participant tends to decrease as the distance from the heart increases. This finding indicates that the proposed method can detect bioelectric activities at a certain distance from the point where a bioelectrode is attached. In consideration of the fact that the conventional method (using a pair of bioelectrodes attached at one measurement point) cannot measure ECG bioelectric signals at the upper or lower arm, this capability is a major feature of the proposed method. As for this feature, as shown in Fig. 4, in the second bioelectric-signal waveform obtained at the upper arm (BB) when the muscles were not moved, ECG potentials generated from the heart and EMG potentials generated from lower-arm muscles both appeared.

**Figure 6:**
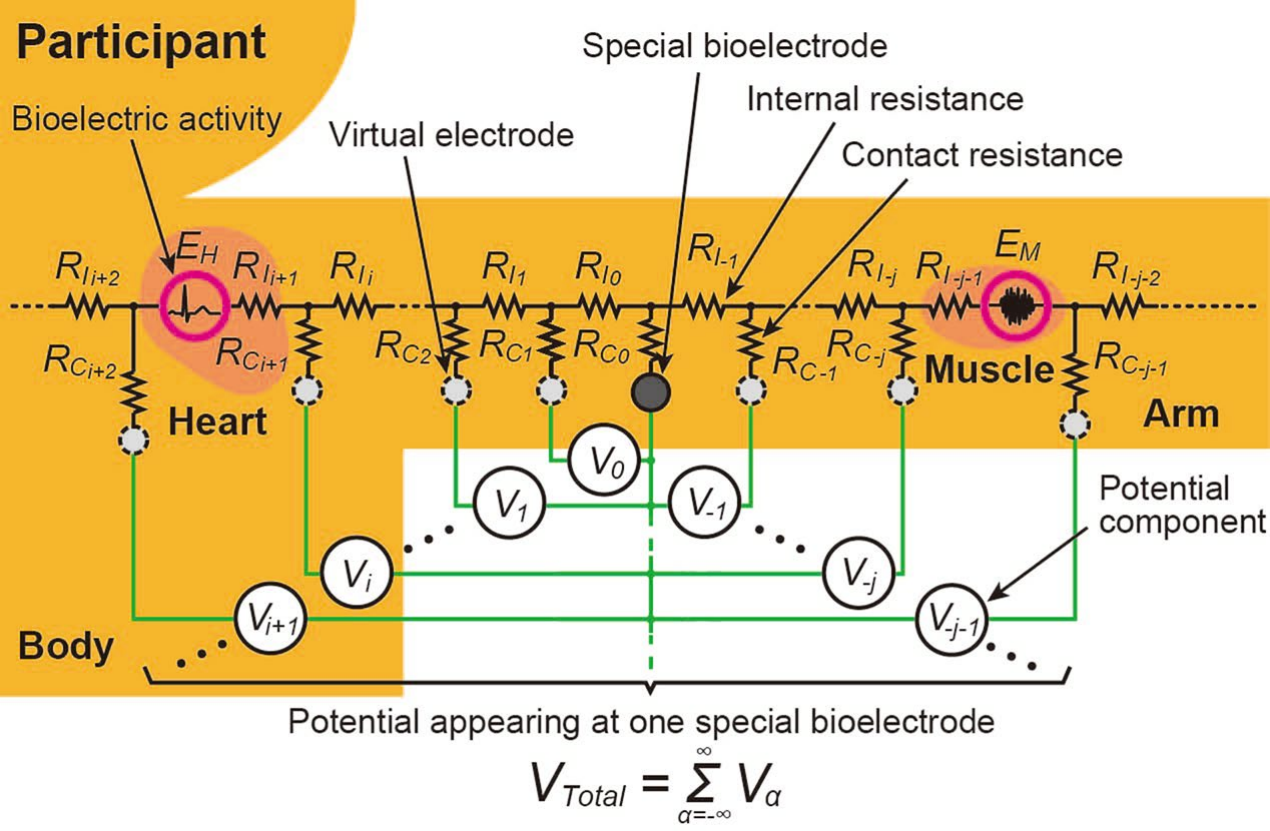
Electric-circuit model of novel method for measuring bioelectric signals in seawater.

The proposed method is based on the bioelectric circuit model shown in Fig. 6. Since the resistance of seawater is extremely low, the bioelectric circuit is modeled under the assumption that the common electrode is a large number of virtual electrodes attached to the surface of a living body. In the bioelectric circuit, internal resistance *ΣR*_*Iα*_ increases with increasing distance between the bioelectric-activity part and the special bioelectrode; thus, potential component *V*_*α*_ generated on the special bioelectrode by bioelectric activity *E*_*α*_ becomes smaller. If bioelectric activities like this occur simultaneously at various parts in the living body, their potential components are added together, and the total potential *V*_*Total*_ (= *ΣV*_*α*_) appears at the special bioelectrode only. This circuit model can therefore be used to explain qualitatively that multiple bioelectric activities at parts away from the special bioelectrode can be observed as shown in the second bioelectric-signal waveform (BB) in Fig. 4.

**Figure 7:**
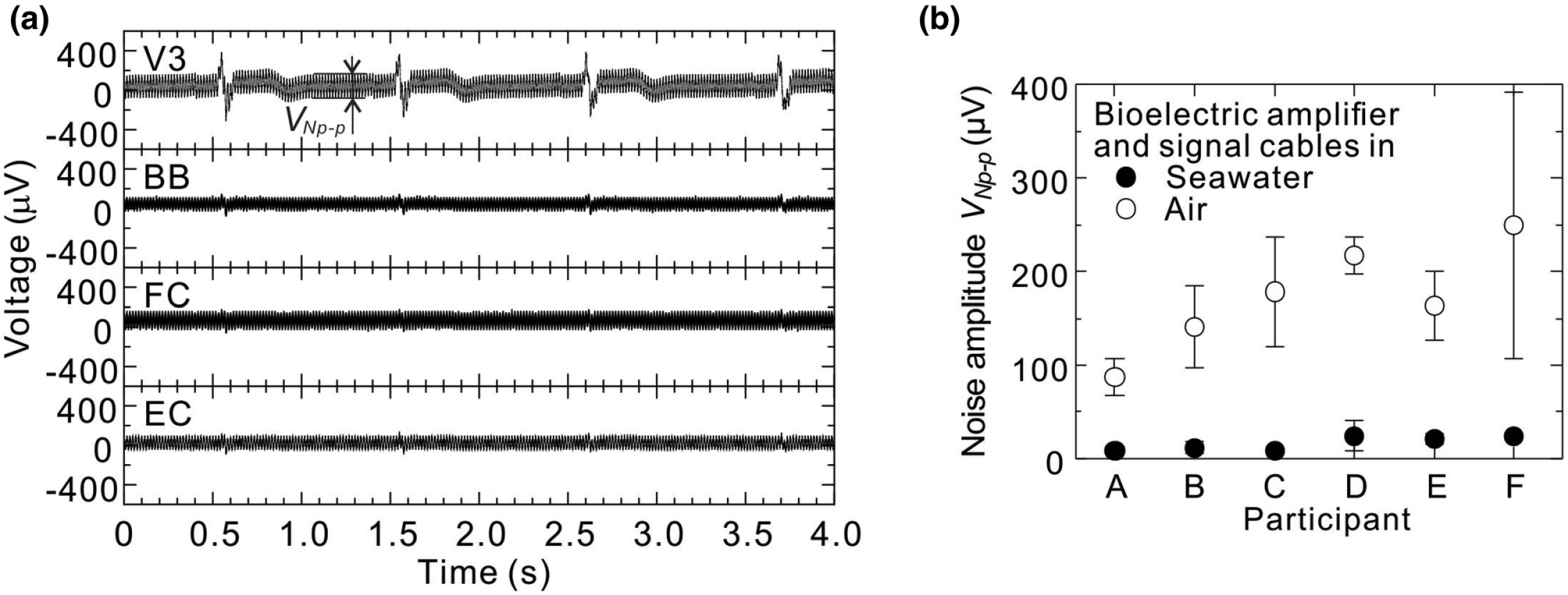
(**a**) Example of bioelectric signals of the participant in rest state when the bioelectric amplifier and signal cables are kept in air (participant A) and (**b**) noise amplitudes included in the bioelectric signals of the participant in rest state. Measurement accuracies of the waveforms at thorax (V3), biceps brachii (BB), flexor carpi (FC), and extensor carpi (EC) were 5.3, 2.6, 2.6, and 2.6 µV, respectively.

Bioelectric signals of participant A at rest, when the bioelectric amplifier and the signal cables were lifted out from the seawater of the bathtub, are shown in Fig. 7a. By visually comparing the bioelectric-signal waveforms in Fig. 7a and those in Fig. 5a, it is clear that at all the measurement points, the noise amplitudes (*V*_*Np-p*_) when the bioelectric amplifier and the signal cables were kept in air are much bigger than those when they were kept in seawater. Incidentally, the noises were hums with commercial frequencies of 60 Hz.

The noise amplitudes in the participants’ resting state when the bioelectric amplifier and the signal cables were kept in air and seawater are summarized for each participant in Fig. 7b. Here, black and white circles represent the average of noise amplitude *V*_*Np-p*_ obtained from four measurement points (V3, BB, FC, and EC) when the bioelectric amplifier and the signal cables were kept in seawater and air, respectively, and the error bars represent their standard deviations. As shown in Fig. 7b, by transferring the bioelectric-measurement system from air to seawater, noise amplitude greatly reduced and became several-dozen microvolts. Incidentally, noise amplitude could be varied easily by changing the signal cable route in air, but it could not be varied in seawater at all. The above-mentioned findings show that by measuring the bioelectric signals in a liquid with high conductivity, the influence of external electrical noise on the measurement system can be greatly reduced.

The proposed method has the two advantages: the bioelectric signals can be measured (i) with fewer bioelectrodes and (ii) in a low-noise environment. However, it is necessary to be careful when using the proposed method for measuring bioelectric signals of a person diving in the actual sea. Specifically, in an area of the sea with low salinity, such as a river mouth, the conductivity of seawater around a diver becomes lower, i.e., the resistance between the special bioelectrode and the common electrode is increased. As a result, the bioelectric signal obtained from the electrodes is reduced. In other words, if the distance between the electrodes becomes shorter, i.e., the resistance between them is decreased, the proposed method can also be used in freshwater on the basis of the same principle as that of the non-isolation method (Fig. 1a in “Introduction”). Incidentally, we experimentally confirmed this phenomenon in a bathtub filled with tap water (17.3 mS/m).

If the proposed method is used for medical diagnoses of patients and evaluating motor skills of athletes, we imagine that the whole body of the patient or athlete will not have to be immersed in seawater. Therefore, when only the lower arm of a subject was immersed in a water tank (60-cm length × 45-cm width × 45-cm height) filled with approximately 95 L of simulant seawater, we measured the bioelectric signals generated from the flexor carpi (FC) and extensor carpi (EC). The result of the measurement showed that two EMG potentials were observed, and the external noise was reduced. From this result, we consider that the advantages of the proposed method are not impaired even in the case of bioelectric measurement in which only a part of a human body is immersed in seawater.

## Conclusion and future work

A method of noninvasive bioelectric measurement was devised by using two kinds of electrodes, one common electrode sunk into seawater covering a person’s entire body and multiple special bioelectrodes isolated from the seawater and attached at various measurement points on the body. An experimental system to test the proposed method was then constructed, and its effectiveness was investigated.

The results of the investigation revealed that using the proposed method enables multipoint bioelectric measurement with about half the number of bioelectrodes used by conventional methods on land, i.e., in air. Moreover, a plurality of bioelectric phenomena may be observed at one measurement point. It was also revealed that compared with the conventional method, the proposed method significantly reduces the external electrical noise included in the bioelectric signals by exploiting the shielding effect of seawater.

In the future, to better understand one bioelectric activity at one measurement point, it is planned to study how to adequately divide the bioelectric signal obtained by the proposed method by signal processing. It is also planned to miniaturize not only the bioelectrodes but also the entire bioelectric-measurement system using the proposed method by applying MEMS (micro electro mechanical systems) and surface-mounting technologies. As a result of that miniaturization, it will be possible to measure bioelectric signals of divers and marine animals in a real marine environment.

## Data Availability

The data that support the findings of this study are available from the corresponding author upon reasonable request.
